# Exercise Training under Exposure to Low Levels of Fine Particulate Matter: Effects on Heart Oxidative Stress and Extra-to-Intracellular HSP70 Ratio

**DOI:** 10.1155/2017/9067875

**Published:** 2017-12-13

**Authors:** Aline Sfalcin Mai, Analu Bender dos Santos, Lílian Corrêa Costa Beber, Renan Daniel Bueno Basso, Lucas Machado Sulzbacher, Pauline Brendler Goettems-Fiorin, Matias Nunes Frizzo, Claudia Ramos Rhoden, Mirna Stela Ludwig, Thiago Gomes Heck

**Affiliations:** ^1^Department of Life Sciences, Research Group in Physiology, Regional University of Northwestern Rio Grande do Sul State (UNIJUÍ), Ijuí, RS, Brazil; ^2^Postgraduate Program in Integral Attention to Health (PPGAIS-UNIJUÍ/UNICRUZ), Ijuí, RS, Brazil; ^3^Laboratory of Oxidative Stress and Air Pollution, Postgraduate Program in Health Sciences, Federal University of Health Sciences of Porto Alegre (UFCSPA), Porto Alegre, RS, Brazil

## Abstract

Fine particulate matter (PM_2.5_) promotes heart oxidative stress (OS) and evokes anti-inflammatory responses observed by increased intracellular 70 kDa heat shock proteins (iHSP70). Furthermore, PM_2.5_ increases the levels of these proteins in extracellular fluids (eHSP70), which have proinflammatory roles. We investigated whether moderate and high intensity training under exposure to low levels of PM_2.5_ modifies heart OS and the eHSP70 to iHSP70 ratio (H-index), a biomarker of inflammatory status. Male mice (*n* = 32), 30 days old, were divided into six groups for 12 weeks: control (CON), moderate (MIT) and high intensity training (HIT), exposure to 5 *μ*g of PM_2.5_ daily (PM_2.5_), and moderate and high intensity training exposed to PM_2.5_ (MIT + PM_2.5_ and HIT + PM_2.5_ groups). The CON and PM_2.5_ groups remained sedentary. The MIT + PM_2.5_ group showed higher heart lipid peroxidation levels than the MIT and PM_2.5_ groups. HIT and HIT + PM_2.5_ showed higher heart lipid peroxidation levels and lower eHSP70 and H-index levels compared to sedentary animals. No alterations were found in heart antioxidant enzyme activity or iHSP70 levels. Moderate exercise training under exposure to low levels of PM_2.5_ induces heart OS but does not modify eHSP70 to iHSP70 ratio (H-index). High intensity exercise training promotes anti-inflammatory profile despite exposure to low levels of PM_2.5_.

## 1. Introduction

Fine particulate matter (PM_2.5_) inhalation promotes cardiovascular injury by direct and/or indirect pathways related to oxidative stress (OS) [1, 2]. The effect of PM_2.5_ is augmented during exercise [3], by increasing deposition in the lungs [4], and may induce lung reflexes which initiate alterations in the autonomic nervous system, increasing heart OS [2]. In this way, under exposure to urban particles or intratracheal administration of a high amount of particles before a moderate intensity exercise session, it was observed that in fact acute exercise in those exposed to particles promotes OS in the heart [1, 5]. Thus, OS induced by particle inhalation may have a negative impact on cardiovascular function and decrease physical performance [6].

The pro-oxidant state of an organism evokes the cell stress response, identified by increasing levels of stress proteins such as the 70 kDa heat shock proteins (HSP70). Intracellularly located HSP70 (iHSP70) maintain homeostasis, by avoiding the formation of toxic polypeptide aggregates that may trigger apoptosis or inflammation [7]. Also, OS is related to alterations in HSP70 release to the extracellular space (eHSP70), including under PM_2.5_ exposure [8, 9]. Systemic inflammation and OS induced by PM_2.5_ in the bloodstream [10] may be accompanied by increasing plasma eHSP70 levels [11, 12], suggesting these proteins as biomarkers of environmental air pollution effects. Once secreted, eHSP70 works as a proinflammatory cytokine (or chaperokine) and signals to different organs and cells for the presence of homoeostatic challenges after eHSP70 binding to Toll-like receptors (TLR-2, TLR-4, and TLR-7). This activates NF-*κ*B-centred proinflammatory pathways thereby stimulating the immune system at different key points and eventually triggering proinflammatory responses that elevate immunosurveillance. Also, eHSP72 concentrations positively correlate with markers of chronic inflammation in humans, for example, C-reactive protein, monocyte count, and TNF-*α* [13–15]. Also, since eHSP70 activate proinflammatory pathways associated with OS conditions [13, 14] and plasma eHSP70 levels are correlated with cardiovascular complications and the severity of cardiac diseases [16], eHSP70 detection reflects both a proinflammatory biomarker and a signalling pathway in progress.

On the other hand, increasing eHSP70 levels are related to the intensity of acute exercise performed in humans [17] and animal models [18] and may participate in fatigue perception by “chaperokine” action of eHSP70 [13]. Also, failure of cell stress response observed by heat shock response impairment [14] supports the relation between exercise and immunoinflammatory status, the predicted “inverted U” relationship (or the “U-shaped” association between exercise and resistance to disease) [17]. Although exercise induces the release of eHSP70 and elevates immunosurveillance by triggering a proinflammatory response [18], high levels of eHSP70, simultaneously with an oxidative profile, may be considered critical for homeostatic status [8]. Thus, since eHSP70 have proinflammatory roles and iHSP70 the opposite, the extra-to-intracellular HSP70 ratio (a.k.a. H-index of HSP70 status) has previously been related to the immune inflammatory balance in exercise and in PM_2.5_ exposure experiments separately [9, 14, 18]. Chronic effects of exercise (training) at different intensities (representing guideline recommendations to improved cardiovascular health that preconizes moderate or vigorous exercise weekly to improve global health) on eHSP70 levels or H-index (and its relation with OS induced by environmental pollution) have been poorly studied. Since alterations in OS, eHSP70, and H-index can mark the subclinical process of tissue and systemic injury, we investigated whether exercise training under exposure to low levels of PM_2.5_ induces heart OS and stress protein (H-index) imbalance.

## 2. Materials and Methods

### 2.1. Animals and Experimental Design

Male (*n* = 32) 30-day-old B6.129SF2/J mice (approximately 18 g) from the Animal Facility of the Regional University of Northwestern Rio Grande do Sul State (UNIJUÍ) were kept in semimetabolic cages, under controlled conditions of temperature (22 ± 2°C) and light-dark cycles (light from 7:00 a.m. to 7:00 p.m.). The animals received water and diet ad libitum. This protocol was approved by the Animal Ethics Committee of UNIJUÍ (CEUA 011/13).

The experimental design that characterizes the periodized exercise training and subchronic PM_2.5_ exposure protocol is summarized in Table 1.

Mice were randomly divided into six treatment groups for 12 weeks: control (CON), moderate intensity exercise training (MIT), high intensity exercise training (HIT) (*n* = 5 per group), exposed to PM_2.5_ (PM_2.5_), and moderate and high intensity training with PM_2.5_ exposure (MIT + PM_2.5_ and HIT + PM_2.5_, resp., *n* = 6 per group). The PM_2.5_, MIT + PM_2.5_, and HIT + PM_2.5_ groups received intranasal instillation of PM_2.5_ (5 *μ*g·10 *μ*L^−1^) once daily, immediately before each exercise session, when applicable. The control, MIT, and HIT groups received 10 *μ*L of saline solution. All groups received their respective nasotropic treatment daily for 12 weeks. The experimental design of PM_2.5_ treatments characterizes a subchronic PM_2.5_ exposure protocol, defined as the repeated exposure by inhalation route for more than 30 days up to 90 days in typically used laboratory animal species (as followed in https://ofmpub.epa.gov, Terminology Service by the United States Environmental Protection Agency).

Exercise training protocols were applied to separate groups in three conditions: sedentary, moderate intensity training, and high intensity training, performed in the following groups: control (CON: remained sedentary), moderate intensity training (MIT: periodized swimming exercise training, for 60 min, 5x/wk, 4% overload), and high intensity training (HIT: periodized swimming exercise training, for 20 min, 5x/wk, 8% overload). Groups exposed to PM_2.5_ received daily nasotropic instillation of 5 *μ*g of PM_2.5_ and remained sedentary (PM_2.5_ group) or were submitted to moderate or high intensity training (MIT + PM_2.5_ and HIT + PM_2.5_ groups, resp.).

All animals were accustomed to the water environment prior to exercise to avoid any stress response related to the new environment and situation. The adaptation period consisted of keeping the animals for 8 min in individual swimming pool chambers (10 cm × 10 cm × 30 cm) filled with water at 31 ± 1°C (20 cm depth), for three consecutive days, without any overload, to avoid stress behaviour (dive and freeze) during exercise training sessions. Afterwards, animals were randomly assigned to each training intensity protocol according to the exercise burden imposed by a progressive lead overload (until 4% or 8% of body weight) attached to the base of the tail and were then submitted to swimming exercise. Individual swimming pool chambers (20 cm deep) avoid jump and dive behaviour and allow energy expenditure higher than 3 METs [19]. Swimming experiments were always carried out between 1:00 and 3:00 p.m. Room temperature during the experiments was kept at 24°C. All the procedures were in accordance with those prescribed in The American Physiological Society's *Resource Book for the Design of Animal Exercise Protocols* [19], and an experienced researcher was present at all times to prevent drowning. Sedentary animals (CON and PM_2.5_ groups) remained at rest in shallow water (2 cm depth; 31 ± 1°C, 20 min) during each training session. A swimming time of 20 min was chosen for the HIT groups because this is the time limit within which an animal really swims with 8% workload. Hot (42°C) and cold (20°C) water may promote hyperthermia and hypothermia in rodents, and many authors suggest that 30°C is a good environment for swimming for Wistar rats and mice (for review see [19]). At this temperature, there is no increase in core temperature during exercise [14]. We assessed core temperature of the animals with the Minipa Digital ThermometerMT450 equipped with a rectal probe. As depicted in Materials and Methods, before exercise, averaged animal core temperature was 36.5 ± 1.22°C and decreased to 32.5 ± 0.8°C after 20 min in all test animals (*P* = 0.008, paired *t-*test). However, it was observed that there was no temperature difference between test groups (*P* = 0.897, ANOVA), so any heat shock response modification observed in our work was induced by exercise or PM_2.5_ and did not represent a heat stress or psychological stress situation. The animals were killed by decapitation 48 h after the last exercise session to obtain blood samples and for heart extraction. Since acute exercise effects may increase oxidative stress markers and eHSP70 release above resting levels during and immediately after exercise and since iHSP70 concentration also increases after exercise and returns to basal levels in 24–48 h later, this 48 h delay period for sample collection was chosen to avoid acute effects of exercise (under PM_2.5_ exposure) that could mask oxidative and/or inflammatory effects of subchronic PM_2.5_ exposure associated to exercise training.

### 2.2. Characterization of Particulate Matter

The pollutant used in the experiment was PM_2.5_, collected in a polycarbonate filter through a gravimetric collector, on the terrace of the Faculty of Medicine, University of São Paulo (USP) in São Paulo, Brazil, as previously described [20]. The exposure site was located close to a monitoring station of the State of São Paulo Sanitation Agency. It is estimated that at least 100,000 vehicles circulate daily on the main and lateral street (~83% cars, ~10% diesel vehicles, and ~6% motorcycles). There are no industries or significant biomass sources in the surrounding area. Trace element determinations of PM_2.5_ content were carried out by neutron activation analysis, and their concentrations were as follows: As = 12.91 ± 0.53; Br = 8.88 ± 0.39; Co = 1.14 ± 0.04; La = 2.33 ± 0.29; Mn = 27.5 ± 2.2; Sb = 8.73 ± 0.08; Sc = 0.141 ± 0.009; Th = 0.351 ± 0.50 (expressed as ng·m^−3^ of air), and Fe = 8.88 ± 0.39 and Cl = 7.85 ± 0.32 (expressed as *μ*g·m^−3^ of air). Likewise, the PM sulfur concentration, determined by X-ray fluorescence analysis, was 1.424 ± 0.08 *μ*m·m^−3^. Briefly, after exposure (24 h), the filter was removed and retained; particles were obtained by sonication, with an ultrasound bath in seven sessions (50 min each), and resuspended in saline solution at a dose of 5 *μ*g·10 *μ*L^−1^. The process of nasotropic instillation was performed once a day (at 1:00 and 2:00 p.m.), immediately before each exercise session, when applicable, for 12 weeks with an automatic pipette, with 10 *μ*L of solution in the nostril of the animal. This procedure induces an apnoea reflex promoting inhalation of the pollutant. The dose used (5 *μ*g, intranasal) represents a low dose of particle exposure [9], since the primary ambient air quality was standard for PM_2.5_, defined by an annual arithmetic mean of 12 *μ*g PM_2.5_·m^−3^ (https://www.epa.gov/pm-pollution).

Our protocol with 5 *μ*g/daily represents an exposure to an urban environment with annual average PM_2.5_ approximately 5 *μ*g/m^3^. Also, it is estimated that 2.5 *μ*m particles have deposition close to 50% in nasal, pharyngeal, and laryngeal areas, while 33–50% of particles of PM_2.5_ may reach alveolar spaces after intranasal administration, and that particles between 0.1 and 0.001 *μ*m may translocate to extrapulmonary organs via the blood compartment. To our knowledge, this is the lowest dose PM_2.5_ exposure protocol used in experimental models [9] and represents an exposure level below alert recommendation levels.

### 2.3. Body Weight, Blood Lactate, and Fasting Glycaemia

The biometric profile was monitored before the randomization, at the 4th, 8th, and 12th weeks. Body weight was checked with a semianalytical scale. Caudal venous lactate concentrations (~25 *μ*L of blood) were determined after exercise in the 5th and 9th weeks by a lactate analyser (Accutrend®Plus System, Roche). The results were expressed as mmol·L^−1^. Blood glucose was evaluated before the randomization, at the 4th, 8th and 12th week after 12 h of fasting. Blood glucose was measured by an Optium Xceed Glucometer (Abbott) (5 *μ*L of blood), by puncturing the distal part of the tail of mice. The glycaemia results were expressed in mg·dL^−1^.

### 2.4. Tissue Preparation

At the end of the 12 weeks of intervention, the animals were euthanized. Hearts were dissected, weighed, freeze-clamped in liquid nitrogen, and stored for further homogenization and analysis of antioxidant activity of the enzymes superoxide dismutase (SOD) and catalase (CAT) and lipid peroxidation levels. A portion of the tissues was homogenized in potassium phosphate buffer pH 7.4 containing the protease inhibitor PMSF (phenylmethylsulfonyl fluoride, 100 *μ*M). Afterwards, the homogenates were centrifuged at 1200 ×g for 10 min at room temperature and the supernatant fractions were saved for protein determination by the spectrophotometric method [21] at 595 nm, using bovine serum albumin as standard.

### 2.5. Lipid Peroxidation

Homogenates were precipitated with 10% trichloroacetic acid (TCA), centrifuged, and incubated with thiobarbituric acid (1 : 1 *v*/*v*) (T5500, Sigma) for 15 min at 100°C. TBARS were extracted using butanol (1 : 1 *v*/*v*). After centrifugation, the absorbance of the butanol layer was measured at 535 nm. The amount of TBARS formed was expressed in nanomoles of malondialdehyde per milligram of protein (nmol MDA·mg prot^−1^). MDA standard was prepared from 1,1,3,3-tetramethoxypropane (Fluka, USA).

### 2.6. Determination of SOD and CAT Activity

SOD activity was determined by inhibition of autoxidation of pyrogallol [22]. Briefly, in a cuvette, 970 *μ*L of 50 mM Tris/1 mM EDTA buffer (pH 8.2), 4 *μ*L of CAT (30 *μ*M), and 10 *μ*L of homogenate were added and mixed. After that, pyrogallol (24 mM in 10 mM HCl) was added and SOD activity determined at 25°C in a spectrophotometer (420 nm) for 120 s. Results were expressed in units of SOD per milligram of protein (U SOD·mg prot^−1^).

In a quartz cuvette, 10 *μ*L of homogenate and 955 *μ*L of phosphate buffer (50 mM, pH 7.0) were mixed and, after that, 35 *μ*L of hydrogen peroxide (0.01 M) was added and mixed. The decomposition of hydrogen peroxide by CAT activity was determined at 25°C in a spectrophotometer (240 nm) for 120 s [23]. The results were expressed in units of CAT per milligram of protein (U CAT·mg prot^−1^).

### 2.7. Plasma eHSP70 Concentration

Animals were decapitated 48 h after the last exercise session, and whole blood was collected in EDTA-treated tubes. The samples were then immediately centrifuged (2000 ×g, at room temperature for 15 min) to obtain plasma samples. The HSP70 plasma concentration (eHSP70) was measured by using a high-sensitivity HSPA1A-specific HSP70 ELISA Kit (ENZO Life Sciences, EKS-715) in diluted (1 : 4) plasma samples as recommended. Absorbance was measured at 450 nm, and a standard curve constructed from known dilutions of recombinant 72 kDa heat shock protein (HSP72) to allow quantitative assessment of eHSP70 plasma concentration. Quantification was done using a microplate reader (Mindray MR-96A). The intra-assay coefficient of variation was identified as being <2%. It is expected that both the inducible HSPA1A and HSPA6 (HSP70B) forms as well as the cognate HSPA8 form of HSP70 (73 kDa heat shock proteins (HSP73)) should be delivered into the extracellular space of different cell types after appropriate stressful conditions. However, only HSPA1A ELISA kits have been sufficiently tested worldwide and proved to be of enough sensitivity (pg·mL^−1^ range) to detect minute HSP70 quantities in culture media and sera. Additionally, previous results [18] have indicated that the principal eHSP70 forms (HSPA1A and HSPA8) are secreted in similar amounts. Therefore, it was assumed that HSPA1A (eHSP72) is a representative of total eHSP70 secretion.

### 2.8. Haematological Analysis

After decapitation, blood was then immediately collected into heparinized (30 IU·mL^−1^ final volume) tubes (for metabolite measurements) or in disodium EDTA-treated tubes (2 mg·mL^−1^ final volume). Haematological parameters were investigated in EDTA samples in a Horiba ABX Micros 60 haematology analyser (for quantitative cell analysis).

### 2.9. Statistical Analysis

Statistical analysis was developed first using two-way analysis of variance (2-way ANOVA) for the analysis of PM_2.5_ exposure, exercise training, and interaction effects. Post hoc multiple comparisons among groups were performed with Tukey's test. All statistical analyses were performed using GraphPad Prism 6.0 for Windows. The level of significance was set to *P* < 0.05. Results were expressed as mean ± SEM.

## 3. Results

### 3.1. Effects of Exercise Training Intensity under Exposure to Low Levels of PM_2.5_ on Biometrical, Biochemical, and Haematological Parameters

The body weight profile of mice (Table 2) during the experimental period was assessed every 4 weeks. All groups increased body weight from basal data (before treatments) to the end of the experimental period (12th week), with no influence of PM_2.5_ exposure or exercise by training exposed to PM_2.5_ (interaction) (Table 2).

Animals were submitted to 12 weeks of periodized exercise training (5x/wk) at moderate or high intensity and were exposed to low levels of PM_2.5_ daily. Blood lactate concentration was measured to confirm different exercise intensities and evaluated whether PM_2.5_ exposure influences exercise response. In the 5th week of training, when all exercised groups reached 4% of workload (see exercise training protocols in Table 1), blood lactate concentrations were close to 4.0 mmol·L^−1^ in all exercised groups, with no influence of PM_2.5_ exposure (Table 3). When groups performed different exercise training intensities (9th week), blood lactate concentration values were higher in the HIT and HIT + PM_2.5_ groups than in the MIT and MIT + PM_2.5_ groups (Table 3) with no influence of PM_2.5_ exposure.

Fasting glycaemia of mice (Table 4) was assessed every 4 weeks and showed no influence of PM_2.5_ exposure or exercise by training exposed to PM_2.5_ (interaction) (Table 4). At the end of the experimental period, blood samples were collected for haematological profile analysis and were found to have a decrease in monocyte and granulocyte counts in the PM_2.5_ group in comparison with the CON group (Table 5).

### 3.2. Exercise Training under Exposure to Low Levels of PM_2.5_ Increased Heart OS

**Figure 1:**
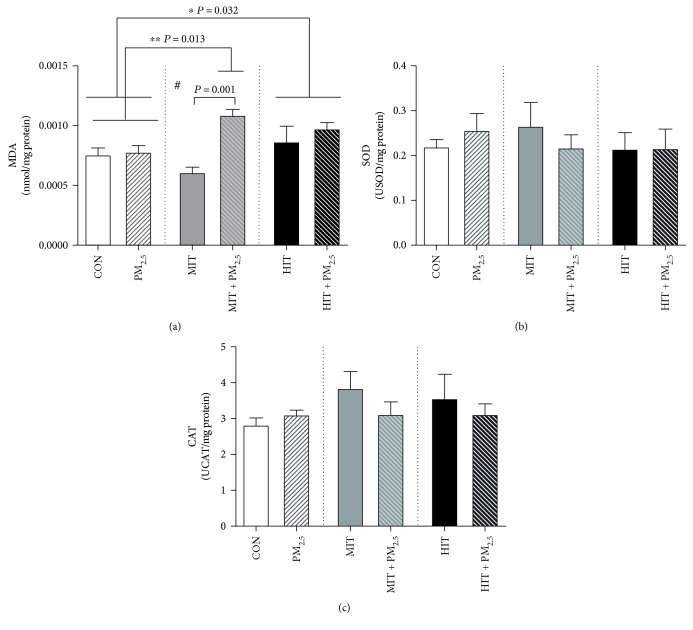
Effect of 12 weeks of exercise training combined with PM_2.5_ exposure on heart oxidative stress profile in mice. Lipid peroxidation levels (a), superoxide dismutase (b), and catalase activity (c) in the heart. CON = control, maintained sedentary. MIT = moderate intensity training. HIT = high intensity training. PM_2.5_ = exposed to PM_2.5_, maintained sedentary. MIT + PM_2.5_ = moderate intensity training exposed to PM_2.5_. HIT + PM_2.5_ = high intensity training exposed to PM_2.5_. PM_2.5_ groups received 5 *μ*g of PM_2.5_ daily (nasotropic) while respective controls received saline. Data analysed by two-way ANOVA followed by Tukey's post hoc test and expressed as mean ± SEM (*n* = 5-6 per group). Lipid peroxidation levels (a): PM_2.5_ effect (*P* = 0.0006), exercise effect (*P* = 0.170), and interaction (*P* = 0.012). Post hoc analyses: ^∗^HIT and HIT + PM_2.5_ versus CON and PM_2.5_ groups (*P* = 0.032), ^∗∗^MIT + PM_2.5_ versus CON and PM_2.5_ groups (*P* = 0.013), and ^#^MIT + PM_2.5_ versus MIT group (*P* = 0.001). Superoxide dismutase activity (b): PM_2.5_ effect (*P* = 0.811), exercise effect (*P* = 0.334), and interaction (*P* = 0.078). Catalase activity (c): PM_2.5_ effect (*P* = 0.339), exercise effect (*P* = 0.472), and interaction (*P* = 0.488).

We investigated heart lipid peroxidation levels and antioxidant enzyme activity to provide information about the effects of exercise training while exposed to low levels of PM_2.5_ on OS markers. Our data showed effects of PM_2.5_ exposure (*P* = 0.0006) and interaction between PM_2.5_ exposure and exercise training (*P* = 0.012) in increasing lipid peroxidation levels in the heart (Figure 1(a)). High intensity exercise per se increased heart lipid peroxidation levels when compared to sedentary mice, independently of PM_2.5_ exposure (*P* = 0.032) (Figure 1(a)). However, the MIT + PM_2.5_ group had increased heart lipid peroxidation levels in comparison with the unexposed group (MIT group, *P* = 0.001) and sedentary mice (CON and PM_2.5_ groups, *P* = 0.013) (Figure 1(a)). The antioxidant activity of heart SOD (Figure 1(b)) and CAT activity (Figure 1(c)) was not modified.

### 3.3. High Intensity Exercise Training Promotes Anti-Inflammatory Profile, Independently of Exposure to PM_2.5_

**Figure 2:**
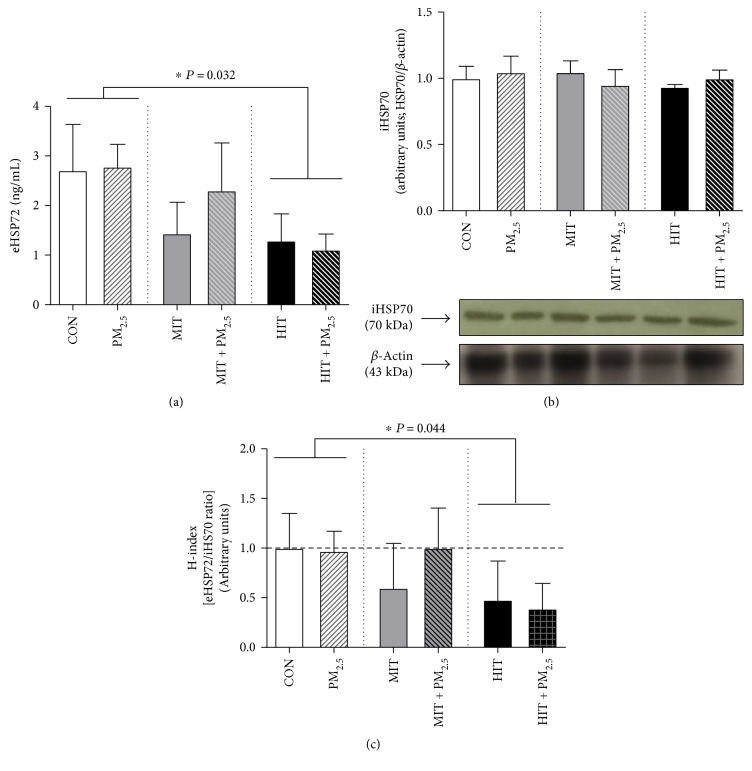
Effect of 12 weeks of exercise training combined with PM_2.5_ exposure on plasma eHSP70, heart iHSP70, and extra-to-intracellular HSP70 ratio (H-index) levels in mice. Plasma eHSP70 (a), heart iHSP70 (b), and extra-to-intracellular HSP70 ratio (H-index) (c) levels. CON = control, maintained sedentary. MIT = moderate intensity training. HIT = high intensity training. PM_2.5_ = exposed to PM_2.5_, maintained sedentary. MIT + PM_2.5_ = moderate intensity training exposed to PM_2.5_. HIT + PM_2.5_ = high intensity training exposed to PM_2.5_. PM_2.5_ groups received 5 *μ*g of PM_2.5_ daily (nasotropic) while respective controls received saline. Data analysed by two-way ANOVA followed by Tukey's post hoc test and expressed as mean ± SEM (*n* = 5-6 per group). eHSP70 levels (a): PM_2.5_ effect (*P* = 0.436), exercise effect (*P* = 0.032), and interaction (*P* = 0.436). Post hoc analyses: ^∗^HIT and HIT + PM_2.5_ versus CON and PM_2.5_ (*P* = 0.032). iHSP70 heart levels (b): PM_2.5_ effect (*P* = 0.952), exercise effect (*P* = 0.827), and interaction (*P* = 0.628). Extra-to-intracellular HSP70 ratio (H-index) levels (c): PM_2.5_ effect (*P* = 0.425), exercise effect (*P* = 0.044), and interaction (*P* = 0.473). ^∗^HIT and HIT + PM_2.5_ versus CON and PM_2.5_ (*P* = 0.044).

Blood samples were collected to evaluate plasma eHSP70 levels as a proinflammatory biomarker. The levels of eHSP70 were not influenced by PM_2.5_ exposure (*P* = 0.672) or by interaction between exercise training and PM_2.5_ exposure (*P* = 0.783) (Figure 2(a)). However, the HIT and HIT + PM_2.5_ groups had decreased eHSP70 levels (*P* = 0.032) in comparison to sedentary animals (CON and PM_2.5_) (Figure 2(a)). There were no alterations in heart iHSP70 levels (Figure 2(b)). Also, low levels of PM_2.5_ exposure did not modify the inflammatory profile observed by the extra-to-intracellular HSP70 ratio (H-index), as observed by similar H-index values (close to 1.0) in CON and PM_2.5_ (Figure 2(c)). However, the H-index in the HIT and HIT + PM_2.5_ groups decreased by approximately by half (H-index = 0.48 and 0.39 in the HIT and HIT + PM_2.5_ groups, resp.; *P* = 0.044) (Figure 2(c)), despite PM_2.5_ exposure. The summary of effects of 12 weeks of exercise training combined with low levels of PM2.5 exposure is expressed in Table 6.

## 4. Discussion

Exercise training can promote countless benefits to the body with highlighted effects on the cardiovascular system. The volume and intensity employed in exercise prescription are crucial to better results. Exercise clearly provides health benefits to humans that subsist even with short sessions beneath the recommended intensity and duration. However, there is no definitive “dose” of exercise that may positively impact the cardiorespiratory system, especially if exercise is performed under the influence of an inadequate air pollution environment. Thus, we investigated whether a “good dose” of exercise can be changed to a “risk dose” if it is practised while exposed to low levels of environmental air pollution. In this case, the population may not interpret a risk and consider an urban area a safe environment in which to walk or run. The major findings of our study were the following: (a) exercise training, even at low levels of environmental air pollution, may predispose to subclinical heart risk (OS); (b) heart OS mediated by particle exposure can be increased by exercise training; (c) training intensity influences OS per se and HSP balance; and (d) high intensity training can promote an anti-inflammatory stress protein profile despite environmental pollution.

First, it is important to highlight that the majority of experimental studies regarding adverse effects of PM_2.5_ on OS parameters (and others) are conducted with animals under rest conditions and use higher levels of particle exposure. Usually, OS is observed in experimental designs that expose mice or rats to high levels of concentrated particles [2], higher levels of aerosol suspension, or intratracheal particle instillation [1, 20]. In the same strain of mice used herein (B6.129SF2/J), in a similar PM_2.5_ exposure protocol (intranasal instillation of 5 *μ*g PM_2.5_, daily for 12 weeks), no increase in OS was observed in the PM_2.5_ exposure group [9]. Considering the filtration possibility by which mouse upper airways can avoid 50% of particle deposition in alveolar spaces, the dose of 5 *μ*g of PM_2.5_ used herein represents a low level of environmental air pollution exposure. To our knowledge, this is the lowest dose of PM_2.5_ exposure ever used in experimental models and represents an exposure level below alert recommendation levels. Thus, OS can be observed under low levels of PM_2.5_ exposure only if associated with another challenge to the organism, such as exercise effort. During exercise, breath frequency (n·min^−1^) and minute ventilation (mL·min^−1^) increases dependent on exercise intensity, and particle deposition in the lungs may also increase in a workload-dependent way [3]. In comparison to rest conditions, exercise increases particle deposition 4.5-fold in humans [24] and 6.0-fold in rodents [25]. These observations corroborate that exercise training under environmental air pollution exposure may represent at least a subclinical risk, marked by inflammatory and oxidative stress profile, but can turn into a clinical risk for more susceptible subjects as aged and cardiovascular disease patients.

Our study evidenced chronicity effects of interaction between exercise and PM_2.5_ exposure since our data represents 12 weeks of continuous exposure and exercise training. Also, biological samples (heart and blood) were extracted 48 h after the last exercise session and PM_2.5_ administration to avoid acute effects on OS and stress protein levels. A previous study showed that intratracheal particle administration (500 *μ*g of residual oil fly ash) in rats, before an acute exercise session at light/moderate intensity (without loads attached to the tail) with a duration of 20 min, promoted an increase in lipid peroxidation levels and a decrease in antioxidant defence (CAT enzyme activity) in lung tissue [1]. In the heart tissue, the same study showed an increase in lipid peroxidation levels without modification of CAT activity. Under urban particulate air exposure, one exercise session at moderate intensity with a duration of 60 min increased lung lipid peroxidation levels when compared to exercise under filtrated air conditions [5]. Since particle inhalation may cause autonomic nervous system imbalance associated with OS damage in the heart and lungs [2] and exercise intensity has a great impact on autonomic nervous system modulation [26], the sum of these challenges (exercise plus particle inhalation) to the cardiopulmonary system can be related to the heart lipid peroxidation observed in our data.

It may be questioned whether the oxidative effects observed in HIT and HIT + PM_2.5_ were induced by higher total energy expended during exercise than in MIT and MIT + PM_2.5_. Also, it may be questioned whether the oxidative effects observed in MIT + PM_2.5_ (in comparison to MIT) were due to distinct exercise performance. Measurements of blood lactate during exercise provide information concerning the energy necessary for execution effort in the animals of our study. A maximum lactate steady state concentration is postulated between 3% and 6% of workload in 92% of mice [27], and a moderate intensity range of 60–75% of VO_2_max at 4.0–4.6% workloads has been suggested [19]. Additionally, an 8% workload may represent a high intensity swimming exercise estimated as representing more than 90% of VO_2_max [28]. Despite this, the estimated total energy expended by animals from the HIT + PM_2.5_ and HIT groups in each session of the last 4 weeks (20 min × 8% workload, blood lactate concentration close to 5.5 mmol·L^−1^) was lower than the MIT + PM_2.5_ and MIT groups (60 min × 4% workload, blood lactate concentration close to 4.0 mmol·L^−1^). Assuming 4.8 kcal (20 kJ) of energy equivalent of consumed O_2_ and 100 mL·kg^−1^·min^−1^ as mouse VO_2_max [19, 29], the total energy expended in each exercise session in the MIT and MIT + PM_2.5_ groups was between 0.45 and 0.56 kcal (assuming 60 min × 0.025 kg × 60–75 mL·kg^−1^·min^−1^), while in the HIT and HIT + PM_2.5_ groups it was 0.22 kcal (assuming 20 min × 0.025 kg × 90 mL·kg^−1^·min^−1^). Our HIT protocol was performed by the attachment of an overload in the tail (8% of body weight) during swimming exercise session. The fatigability of this protocol was previously tested in mice and rats with this workload. Indeed, this swimming time (20 min) was chosen because this is the time limit within which an untrained animal really swims before learning how to perform bobbing, which is a survival strategy used to conserve energy without doing exercise. On the other hand, with 4% weight attached to the tail, mice can swim 60 minutes or more. For this reason, the workloads used herein characterized adequately two distinct exercise intensities. Although our HIT protocol represents higher exercise intensity (as well as a higher fatigable exercise), our MIT protocol represents a more energy-expensive protocol (higher volume of training). Thus, the main effects observed herein (by HIT protocol) were representatives of a high-intensity exercise training effect, despite low energy expended in each exercise session compared to the MIT groups. In this case, HSP70 response observed in our work supports the hypothesis that exercise intensity is an important modulator of HSP70 levels. Thus, the intensity of exercise training increased heart lipid peroxidation in HIT and HIT + PM_2.5_ in comparison to sedentary animals. Also, the HIT and HIT + PM_2.5_ heart lipid peroxidation levels were similar because exercise intensity may be considered a stronger factor than low levels of PM_2.5_ in terms of influence on oxidative metabolism. In this way, MIT did not promote oxidative effects despite the higher amount of energy expended, but when trained and exposed to PM_2.5_, MIT + PM_2.5_ promoted OS. Exercise-induced oxidative stress and HSP70 response include mode, duration, and intensity of exercise, specific biomarkers chosen, the time course of tissue sampling, and the training status. In this way, the majority of the studies have reported an increase in TBARS and HSP70 following both maximal and submaximal exercises, with values typically returning to baseline within one-hour postexercise while the peak of iHSP70 levels is typically four hours after exercise session returning to preexercise levels 24 or 48 h later [14, 30]. Thus, in our study, we collected samples 48 hours after the last exercise session to avoid acute exercise effects on oxidative or HSP70 response.

In our study, exercise training did not avoid the oxidative effects of pollution. Exercise performed before environmental pollution exposure can provide some protection by reducing minute ventilation and frequency of breaths at rest [31]. At an epidemiological level, habitual exercise may prevent premature death attributable to air pollution that decreases being close to the 2.5% risk of mortality for cardiorespiratory diseases by particulate matter with an aerodynamic diameter of 10 *μ*m or smaller [32]. The benefits of exercise training in promoting anti-inflammatory and antioxidant adaptations are well known, observed by increased anti-inflammatory cytokine levels and antioxidant defences [33], protecting against many different challenges and acting as a “polypill” [34]. These adaptations are able to prevent oxidative and inflammatory challenges that reach a trained organism, such as air pollution challenge itself [31], but may not prevent injury if exercising under PM_2.5_ exposure, with increasing particle deposition.

Exercise intensity has also been studied in terms of stress protein response. Historically, studies have been dedicated to iHSP70 analysis in cardiac or skeletal muscle after exhaustive animal protocols [13]. This acute heat shock response (iHSP70 content) remains increased 24 h after an acute exercise session, according to exercise load. This overexpression of iHSP70 is associated with many cytoprotective effects under physiological conditions, since HSP70 family members act as molecular chaperones. iHSP70 protect cells against lethal damage induced by stress and support folding and transport of newly synthesized polypeptides and aberrant proteins as well as the assembly of multiprotein complexes [35]. In our data, the iHSP70 level was similar among experimental groups due to its measurement 48 h after the last exercise session. Also, the exercised mice had the same intensity and duration of exercise during the last 4 weeks of the protocol (from 9th to 12th weeks), providing cellular adaptation to the same exercise effort.

While iHSP70 represent the intracellular stress response, eHSP70 are considered molecules with immunomodulatory functions, such as chaperokines that can stimulate innate and adaptive immunity [35]. Studies have shown that chronic exposure to air pollutants increases eHSP70 levels in exposed workers and this effect is related to lipid peroxidation [11, 12], suggesting that plasma eHSP70 levels could be used as a sensitive responsive biomarker for environmental challenges. Interestingly, in response to acute exercise, cells can release these proteins to extracellular fluids, and they can be measured in plasma samples [30]. eHSP70 release by lymphocytes and monocytes is inferred from the analysis of heat shock response in an acute exercise session, related to the profile of immune responses [13, 14, 18]. Since eHSP70 exist in the extracellular space as a danger signal from the heat shock response pathway, molecular interactions with cell surface receptors may occur and signalling pathways could be triggered in many cell types, whereas there is a variety of receptors to HSP70 binding, amplifying the possible targets of these extracellular molecules. The potential of the whole organism to efficiently trigger a robust heat shock response has enormous clinical repercussions, with inflammatory-related effects, and these effects are dependent on exercise intensity [14].

Since iHSP70 are anti-inflammatory proteins whose expression is highly depressed in chronic inflammatory conditions, eHSP70 operate in an opposing fashion. In fact, the extra-to-intracellular HSP70 ratio (H-index), measured in different tissues and cell types in relation to plasma or culture media, has started to be ascribed as a novel and overall index of the immunoinflammatory status of an individual [9, 13, 14, 18]. In other words, higher eHSP70 amounts represent more inflammatory signals and may be related to danger or stress physiological markers since the adrenergic response mediated by *α*_1_-adrenoceptors also influences circulating levels of eHSP70, as a general protective role of eHSP70 in warning physiological systems [13] about the presence of homeostasis-threatening situations. Thus, decreased eHSP70 and H-index levels may be a marker of the autonomic modulation effect of high intensity training observed in both the HIT and the HIT + PM_2.5_ groups. Thus, despite environmental pollution, which may impair autonomic function resulting in heart OS [2], exercise training may promote protective and therapeutic effects by having the opposite impact on the autonomic nervous system, by improving vascular function and cardiac remodelling [36]. Because eHSP70 is proinflammatory cytokine in its nature and participates in the local inflammatory process in the heart [16] suggested as a biomarker for early diagnosis of heart failure [37], the state of anti-inflammation indicated in HIT and HIT + PM_2.5_ by reduced H-index [14] indicates the ability to counteract noxious/danger signals and protect chemotaxis signalling to the organ [16] affected by PM_2.5_-induced OS.

Recently, one study showed that exercise training can decrease eHSP70 levels [38] but, to our knowledge, our study is the first evidence suggesting that exercise training is able to reduce the H-index. Since H-index directly correlates with the pro/anti-inflammatory cytokine ratio [14] and there is a positive correlation between eHSP70 levels and the proinflammatory cytokine profile [14, 39, 40], our work reinforces the anti-inflammatory effects of exercise.

Finally, to avoid possible interpretation mistakes in eHSP70 levels and H-index, we evaluated fasting glycaemia, body weight, and haematological parameters. Since obesity, type two diabetes mellitus, and inflammation [9, 40] increase eHSP70 levels, no alterations in fasting glycaemia and body weight exclude the hypothesis of confounding factors that explain the reduction of eHSP70 levels and H-index. Although it is well known that acute particle exposure can stimulate bone marrow to enhance the release of neutrophils and monocytes into the circulation (which facilitates a cellular inflammatory response), there was evidence that PM_2.5_ exaggerated adhesion of monocytes in mesenteric microvessels, culminating in accumulation in visceral adipose [41]. These intriguing findings suggest that longer-term exposure to PM air pollution may promote chronic development of insulin resistance, obesity, and the metabolic syndrome, as recently reported by us [9]. In addition, longer PM_2.5_ inhalation may represent the sequestration of these cells into tissue compartments such as the lung or vasculature, including the recruitment of circulating monocytes into atherosclerotic plaques that may be an important step in the PM_2.5_-induced progression of atherosclerosis [42]. Also, decreases in blood monocytes, basophils, eosinophils, and CD54 and CD18 adhesion molecule expression on monocytes after exposure to ultrafine carbon among exercising asthmatic individuals and healthy adults have also been reported [41]. In both exercise training groups (MIT + PM_2.5_ and HIT + PM_2.5_), these effects (decrease in monocyte and granulocyte count) were not observed, suggesting beneficial effects of exercise in agreement of H-index data.

Our study has some limitations in terms of variables that could allow more detail about cardiovascular function and inflammatory and oxidative markers. Also, it can be pointed that larger sample sizes could possibly show that the activities of heart antioxidant enzymes CAT (*P* = 0.132) and SOD (*P* = 0.071) became statistically different between the MIT and the MIT + PM_2.5_, groups. However, even if antioxidant enzyme activities were now considered to be altered, the oxidative damage observed (lipid peroxidation levels) as an effect of combination MIT + PM_2.5_ still remains unaltered, reinforcing the effects of exercise and PM_2.5_ interaction. Although TBARS assay may be considered an unspecific test, allowing a global approach of lipoperoxidation, other more specific determinations can confirm the end-product formed during oxidative stress. Also, aldehydes other than MDA can form chromogens and many different aldehydes are formed during lipid peroxidation and/or during the acid-heating stage of the TBARS test [43]. Whether H-index might be used as a biomarker of environmental air pollution injury (as it is the case for obesity and T2DM) and if the decrease in H-index following high exercise training might guarantee better cellular protection requires further investigation.

It is important to know that the sum of evidence from epidemiological, clinical, and experimental studies strongly indicates that exercise performance [6] and cardiovascular health are both affected by other air pollutants such as the gaseous pollutants ozone, nitrogen dioxide (NO2), volatile organic compounds (including benzene), carbon monoxide (CO), and sulphur dioxide (SO2) [44]. Also, stress proteins (HSP70 in particular) are suggested to be potentially sensitive indicators of any chemical or physical insult and have been successfully used recently as an early, first tier marker for the assessment of environmental chemicals [9, 45] as well as a biomarker of cardiovascular health status [15, 46]. Thus, new studies to evaluate H-index under exercise/training conditions concomitantly with air pollution exposure represent a promising and extensive field of research in air pollution biomarkers.

## 5. Conclusion

Together, these previous studies and our data indicate that acute and chronic exercise exposed to PM_2.5_, even at low doses of pollution, may be a risk for heart OS. Also, the intensity of exercise training is a critical parameter for exercise prescription under PM_2.5_ exposure to avoid adverse environmental pollution effects on an organism. Moderate exercise training under exposure to low levels of PM_2.5_ induces heart OS but does not modify eHSP70 to iHSP70 ratio (H-index) balance. High intensity exercise training promotes anti-inflammatory profile despite exposure to low levels of PM_2.5_.

## Figures and Tables

**Table 1:** Experimental protocol of swimming exercise training in mice exposed to fine particulate matter (PM_2.5_).

Experimental groups	Periodized swimming exercise training protocols	Fine particulate matter (PM_2.5_) exposition protocol
CON	Mice maintained sedentary: 20 minutes in 30°C shallow water (2 cm), 5x/week, 12 weeks.	Mice received 10 *μ*L of NaCl 0.9% by intranasal instillation daily (7x/week) for 12 weeks
MIT	Mice submitted to moderate intensity swimming training in 20 cm deep water (30°C), 5x/week, 12 weeks. Protocol started with 20 minutes of exercise. In the following weeks, the workload increased by additional weight attached in mice: in the 2nd week it increased to 1%, in the 3rd week to 2%, in the 4th week to 3%, and in the 5th week to 4%. In the following weeks, the session duration increased 10 min/week up to 60 minutes reached in the 9th week. These workload and duration remained unaltered until the 12th week.	Mice received 10 *μ*L of NaCl 0.9% by intranasal instillation daily (7x/week) for 12 weeks
HIT	Mice submitted to high intensity swimming training in 20 cm deep water (30°C), 5x/week, 12 weeks. Protocol started with 20 minutes of exercise. In the following weeks, the workload increased by additional weight attached in mice: in the 2nd week it increased to 1%, in the 3rd week to 2%, in the 4th week to 3%, and in the 5th week to 4%. In the following weeks, the workload increased 1%/week up to 8% workload reached in the 9th week These workload and duration remained unaltered until the 12th week.	Mice received 10 *μ*L of NaCl 0.9% by intranasal instillation daily (7x/week) for 12 weeks
PM_2.5_	Mice maintained sedentary: 20 minutes in 30°C shallow water (2 cm), 5x/week, 12 weeks.	Mice received intranasal instillation of PM_2.5_ (5 *μ*g of PM_2.5_ suspended in 10 *μ*L of NaCl 0.9%) daily (7x/week) for 12 weeks
MIT + PM_2.5_	Mice submitted to moderate intensity swimming training in 20 cm deep water (30°C), 5x/week, 12 weeks. Protocol started with 20 minutes of exercise. In the following weeks, the workload increased by additional weight attached in mice: in the 2nd week it increased to 1%, in the 3rd week to 2%, in the 4th week to 3%, and in the 5th week to 4%. In the following weeks, the session duration increased 10 min/week up to 60 minutes reached in the 9th week. These workload and duration remained unaltered until the 12th week.	Mice received intranasal instillation of PM_2.5_ (5 *μ*g of PM_2.5_ suspended in 10 *μ*L of NaCl 0.9%) daily (7x/week) for 12 weeks
HIT + PM_2.5_	Mice submitted to high intensity swimming training in 20 cm deep water (30°C), 5x/week, 12 weeks. Protocol started with 20 minutes of exercise. In the following weeks, the workload increased by additional weight attached in mice: in the 2nd week it increased to 1%, in the 3rd week to 2%, in the 4th week to 3%, and in the 5th to 4%. In the following weeks, the workload increased 1%/week up to 8% workload reached in the 9th week These workload and duration remained unaltered until 12th week	Mice received intranasal instillation of PM_2.5_ (5 *μ*g of PM_2.5_ suspended in 10 *μ*L of NaCl 0.9%) daily (7x/week) for 12 weeks

**Table 2:** Body weight of mice exposed to fine particulate matter (PM_2.5_) submitted to 12 weeks of exercise training.

	CON	PM_2.5_	MIT	MIT + PM_2.5_	HIT	HIT + PM_2.5_	ANOVA (*P* value)
Before	19.0 ± 3.0	18.5 ± 3.1	18.0 ± 1.8	17.6 ± 2.3	19.0 ± 1.7	19.5 ± 2.2	0.832
4th week	24.6 ± 2.2^†^	24.9 ± 2.1^§^	23.5 ± 2.7^†^	23.1 ± 2.3^†^	24.9 ± 0.5^†^	22.2 ± 4.0	0.414
8th week	27.3 ± 1.8^§^	28.1 ± 2.7^§^	24.7 ± 2.5^∗^	27.1 ± 3.0^§^	25.3 ± 0.9^†^	25.2 ± 3.9^†^	0.214
12th week	28.0 ± 3.6^§^	28.7 ± 2.8^§^	26.7 ± 2.2^∗^	27.9 ± 2.9^§^	26.7 ± 2.5^§^	26.0 ± 3.3^†^	0.650
Δ (12th week − before)	8.9 ± 4.5	9.1 ± 4.4	8.6 ± 3.2	8.8 ± 3.7	8.1 ± 3.1	6.4 ± 4.7	0.896

Body weight (g) expressed as mean ± standard deviation. CON: control group, received 10 *μ*L saline daily, maintained sedentary. MIT: moderate intensity training group, received 10 *μ*L saline daily. HIT: high intensity training group, received 10 *μ*L saline daily. PM_2.5_: exposure group, received 5 *μ*g of PM_2.5_ daily, maintained sedentary. MIT + PM_2.5_: moderate intensity training group, received 5 *μ*g of PM_2.5_ daily. HIT + PM_2.5_: high intensity training group, received 5 *μ*g of PM_2.5_ daily (*n* = 5-6 per group). Data analysed by two-way ANOVA followed by Tukey's multiple comparison test. There was an effect of time (*P* < 0.001) and no effect of treatment (*P* = 0.129) or interaction between time and treatment (*P* = 0.854). Time effects are indicated as ^∗^*P* < 0.05, ^†^*P* < 0.01, and ^§^*P* < 0.001 versus initial body weight (“before” data) in each respective group. The difference between final and initial body weight (Δ body weight 12th week − before) was not different among experimental groups (*P* = 0.911).

**Table 3:** Blood lactate concentration of mice exposed to fine particulate matter (PM_2.5_) submitted to exercise training.

	CON	PM_2.5_	MIT	MIT + PM_2.5_	HIT	HIT + PM_2.5_	ANOVA (*P* value)
5th week	—	—	4.54 ± 0.21	3.90 ± 0.33	4.17 ± 0.22	4.04 ± 0.19	0.299
9th week	—	—	4.20 ± 0.20	4.00 ± 0.20	5.13 ± 0.86^∗^	5.40 ± 0.36^∗^	0.024^∗^

Blood lactate concentration (mmol·L^−1^) expressed as mean ± SEM. CON: control group, received 10 *μ*L saline daily, maintained sedentary. MIT: moderate intensity training group, received 10 *μ*L saline daily. HIT: high intensity training group, received 10 *μ*L saline daily. PM_2.5_: exposure group, received 5 *μ*g of PM_2.5_ daily, maintained sedentary. MIT + PM_2.5_: moderate intensity training group, received 5 *μ*g of PM_2.5_ daily. HIT + PM_2.5_: high intensity training group, received 5 *μ*g of PM_2.5_ daily (*n* = 5-6 per group). Data analysed by two-way ANOVA followed by Tukey's multiple comparison test. There was no effect of PM_2.5_ (*P* < 0.940) and interaction (*P* = 0.617), but there was an effect of exercise (*P* = 0.033). Both HIT and HIT + PM_2.5_ showed higher levels in the 8th week in comparison with the MIT and MIT + PM_2.5_ groups (^∗^*P* = 0.024) and also higher than itself in the 5th week of exercise effort (^∗^*P* = 0.039).

**Table 4:** Fasting glycaemia of mice exposed to fine particulate matter (PM_2.5_) submitted to 12 weeks of exercise training.

	CON	MIT	HIT	PM_2.5_	MIT + PM_2.5_	HIT + PM_2.5_	ANOVA (*P* value)
Before	120.3 ± 6.4	123.6 ± 23.0	127.2 ± 26.2	116.0 ± 22.4	104.8 ± 37.9	119.2 ± 27.4	0.789
4th week	112.5 ± 19.5	96.2 ± 23.9	85.6 ± 13.7^†^	104.8 ± 23.0	104.0 ± 14.8	99.2 ± 16.6	0.328
8th week	102.5 ± 14.7	93.4 ± 14.7	96.0 ± 19.6	94.8 ± 15.2	96.2 ± 15.1	109.4 ± 22.0	0.652
12th week	91.5 ± 21.3	74.0 ± 10.9^§^	80.7 ± 17.0^†^	80.1 ± 8.8^∗^	77.2 ± 15.6	81.6 ± 4.0^∗^	0.458

Fasting glycaemia (mg·dL^−1^) expressed as mean ± standard deviation. CON: control group, received 10 *μ*L saline daily, maintained sedentary. MIT: moderate intensity training group, received 10 *μ*L saline daily. HIT: high intensity training group, received 10 *μ*L saline daily. PM_2.5_: exposure group, received 5 *μ*g of PM_2.5_ daily, maintained sedentary. MIT + PM_2.5_: moderate intensity training group, received 5 *μ*g of PM_2.5_ daily. HIT + PM_2.5_: high intensity training group, received 5 *μ*g of PM_2.5_ daily (*n* = 5-6 per group). Data analysed by two-way ANOVA followed by Tukey's multiple comparison test. There was an effect of time (*P* < 0.0001) and no effect of treatment (*P* = 0.422) or interaction between time and treatment (*P* = 0.791). The time effects are indicated as ^∗^*P* < 0.05, ^†^*P* < 0.01, and ^§^*P* < 0.001 versus initial body weight (“before” data) in each respective group.

**Table 5:** Haematological parameters of mice exposed to fine particulate matter (PM_2.5_) submitted to 12 weeks of exercise training.

	CON	PM_2.5_	MIT	MIT + PM_2.5_	HIT	HIT + PM_2.5_	ANOVA (*P* value)
WBC (10^3^·mm^−3^)	10.8 ± 2.8	6.8 ± 3.1	8.9 ± 3.3	7.2 ± 3.1	7.6 ± 1.2	6.6 ± 0.8	0.146
LYM 10^3^·mm^−3^ (%LYM)	8.96 ± 2.38 (83.0 ± 4.4)	5.86 ± 2.87 (84.5 ± 1.5)	7.56 ± 3.13 (83.4 ± 3.2)	6.85 ± 2.68 (82.3 ± 2.4)	6.51 ± 0.93 (84.5 ± 4.5)	5.46 ± 0.81 (82.0 ± 3.0)	0.214
MON 10^3^·mm^−3^ (%MON)	0.58 ± 0.19 (5.3 ± 0.8)	0.31 ± 0.14^∗^ (5.0 ± 0.9)	0.43 ± 0.12 (5.0 ± 1.2)	0.47 ± 0.17 (5.1 ± 0.8)	0.37 ± 0.06 (5.2 ± 0.9)	0.35 ± 0.06 (5.1 ± 0.8)	0.041^∗^
GRA 10^3^·mm^−3^ (%GRA)	1.34 ± 0.56 (12.5 ± 3.8)	0.63 ± 0.31^∗^ (9.6 ± 2.8)	0.97 ± 0.19 (11.6 ± 3.2)	0.99 ± 0.30 (12.5 ± 2.5)	0.71 ± 0.50 (10.2 ± 4.9)	0.87 ± 0.15 (13.2 ± 3.1)	0.047^∗^
RBC (10^6^·mm^−3^)	10.5 ± 1.1	7.92 ± 0.4	9.7 ± 1.3	8.94 ± 0.9	7.8 ± 1.7	8.31 ± 0.6	0.220
HGB (g·dL^−1^)	17.4 ± 3.9	13.6 ± 1.5	15.8 ± 4.0	14.6 ± 2.9	13.3 ± 5.1	13.2 ± 2.2	0.286
HCT (%)	40.2 ± 1.5	34.5 ± 1.5	39.8 ± 1.5	40.6 ± 4.5	37.1 ± 1.6	37.6 ± 2.9	0.291
PLT (10^3^·mm^−3^)	1436 ± 329	975 ± 372	1124 ± 364	1157 ± 248	1036 ± 575	906 ± 145	0.210
NEU/LYM ratio (10^3^·mm^−3^)	0.15 ± 0.05	0.11 ± 0.03	0.14 ± 0.04	0.15 ± 0.03	0.10 ± 0.06	0.16 ± 0.04	0.425

Haematological parameters expressed as mean ± SEM. CON: control group, received 10 *μ*L saline daily, maintained sedentary. MIT: moderate intensity training group, received 10 *μ*L saline daily. HIT: high intensity training group, received 10 *μ*L saline daily. PM_2.5_: EXPOSURE group, received 5 *μ*g of PM_2.5_ daily, maintained sedentary. MIT + PM_2.5_: moderate intensity training group, received 5 *μ*g of PM_2.5_ daily. HIT + PM_2.5_: high intensity training group, received 5 *μ*g of PM_2.5_ daily (*n* = 5-6 per group). Data analysed by two-way ANOVA followed by Tukey's post hoc test. *P* values describe here in each variable in terms of the PM_2.5_ effect, exercise effect, and interaction, respectively. WBC: white blood cell (*P* = 0.291, *P* = 0.139, and *P* = 0.106). %LYM: lymphocyte percentage (*P* = 0.629, *P* = 0.548, and *P* = 0.214). %MON = monocyte percentage (*P* = 0.680, *P* = 0.575, and *P* = 0.220). %GRA: granulocyte percentage (*P* = 0.910, *P* = 0.985, and *P* = 0.761). RBC: red blood cells (*P* = 0.567, *P* = 0.783, and *P* = 0.741). HGB: haemoglobin (*P* = 0.118, *P* = 0.777, and *P* = 0.931). HCT: haematocrit (*P* = 0.438, *P* = 0.367, and *P* = 0.291). PLT: platelet total count (*P* = 0.0810, *P* = 0.335, and *P* = 0.224). ^∗^*P* < 0.05 versus the control group; one-way ANOVA.

**Table 6:** Summary of effects of 12 weeks of exercise training combined with low levels of PM_2.5_ exposure.

	Sedentary + low levels of PM_2.5_	Moderate intensity exercise training + low levels of PM_2.5_	High intensity exercise training + low levels of PM_2.5_	Observed effects
Heart oxidative stress	—	Increase^∗^^#^	Increase^∗^	^∗^Compared to sedentary animals exposed or not to PM_2.5_ ^#^Compared to mice exercised without PM_2.5_ exposure
eHSP70/iHSP70 (H-index) inflammatory state	—	—	Decrease^∗∗^	^∗∗^Compared to sedentary animals exposed or not to PM_2.5_
Leucocyte count (granulocytes and monocytes)	Decrease^##^	—	—	^##^Compared to sedentary animals not exposed to PM_2.5_

## Abbreviations

CAT: Catalase
HSP70: 70 kDa family of heat shock proteins
eHSP70: Extracellular HSP70
iHSP70: Intracellular HSP70
H-index: eHSP70 to iHSP70 ratio
OS: Oxidative stress
PM_2.5:_: Fine particulate matter
SOD: Superoxide dismutase
TCA: Trichloroacetic acid.

## References

[1] Heck T. G., Nunes R. B., Petry M. R. (2015). Residual oil fly ash (ROFA) inhalation promotes lung and heart oxidative stress without hemodynamic effects in exercising rats. *Journal of Exercise Physiology Online*.

[2] Rhoden C. R., Wellenius G. A., Ghelfi E., Lawrence J., Gonzalez-Flecha B. (2005). PM-induced cardiac oxidative stress and dysfunction are mediated by autonomic stimulation. *Biochimica et Biophysica Acta (BBA)-General Subjects*.

[3] Sharman J. E., Stowasser M. (2009). Australian association for exercise and sports science position statement on exercise and hypertension. *Journal of Science and Medicine in Sport*.

[4] Rundell K. W., Slee J. B., Caviston R., Hollenbach A. M. (2008). Decreased lung function after inhalation of ultrafine and fine particulate matter during exercise is related to decreased total nitrate in exhaled breath condensate. *Inhalation Toxicology*.

[5] Heck T. G., Petry M. R., Maslinkiewicz A. (2015). Effects of ambient particles inhalation on lung oxidative stress parameters in exercising rats. *Journal of Exercise Physiology Online*.

[6] Florida-James G., Donaldson K., Stone V. (2004). Athens 2004: the pollution climate and athletic performance. *Journal of Sports Sciences*.

[7] Newsholme P., de Bittencourt P. I. (2014). The fat cell senescence hypothesis: a mechanism responsible for abrogating the resolution of inflammation in chronic disease. *Current Opinion in Clinical Nutrition and Metabolic Care*.

[8] Gelain D. P., de Bittencourt Pasquali M. A., MC C. (2011). Serum heat shock protein 70 levels, oxidant status, and mortality in sepsis. *Shock*.

[9] Goettems-Fiorin P. B., Grochanke B. S., Baldissera F. G. (2016). Fine particulate matter potentiates type 2 diabetes development in high-fat diet-treated mice: stress response and extracellular to intracellular HSP70 ratio analysis. *Journal of Physiology and Biochemistry*.

[10] Yamawaki H., Iwai N. (2006). Mechanisms underlying nano-sized air-pollution-mediated progression of atherosclerosis: carbon black causes cytotoxic injury/inflammation and inhibits cell growth in vascular endothelial cells. *Circulation Journal*.

[11] Yang X., Zheng J., Bai Y. (2007). Using lymphocyte and plasma Hsp70 as biomarkers for assessing coke oven exposure among steel workers. *Environmental Health Perspectives*.

[12] Xia B., Chen K., Lv Y. (2017). Increased oxidative stress and plasma Hsp70 levels among gasoline filling station attendants. *Toxicology and Industrial Health*.

[13] Heck T. G., Scholer C. M., de Bittencourt P. I. (2011). HSP70 expression: does it a novel fatigue signalling factor from immune system to the brain?. *Cell Biochemistry and Function*.

[14] Heck T. G., Scomazzon S. P., Scholer C. M., Homem de Bittencourt P. I. (2017). Acute exercise boosts cell proliferation and the heat shock response in lymphocytes: correlation with cytokine production and extracellular-to-intracellular HSP70 ratio. *Cell Stress Chaperones*.

[15] Miragem A. A., Homem de Bittencourt P. I. (2017). Nitric oxide-heat shock protein axis in menopausal hot flushes: neglected metabolic issues of chronic inflammatory diseases associated with deranged heat shock response. *Human Reproduction Update*.

[16] Cai W. F., Zhang X. W., Yan H. M. (2010). Intracellular or extracellular heat shock protein 70 differentially regulates cardiac remodelling in pressure overload mice. *Cardiovascular Research*.

[17] Walsh N. P., Gleeson M., Shephard R. J. (2011). Position statement. Part one: immune function and exercise. *Exercise Immunology Review*.

[18] Scholer C. M., Marques C. V., da Silva G. S., Heck T. G., de Oliveira Junior L. P., Homem de Bittencourt P. I. (2016). Modulation of rat monocyte/macrophage innate functions by increasing intensities of swimming exercise is associated with heat shock protein status. *Molecular and Cellular Biochemistry*.

[19] Kregel K. C., Allen D. L., Booth F. W. (2006). Resource book for the animal exercise protocols. *American physiological Society*.

[20] Maatz L. F., Wood G. J., Rivero D. H., Saldiva P. H. (2009). Tracheal instillation of urban PM_2.5_ suspension promotes acute cardiac polarization changes in rats. *Brazilian Journal of Medical and Biological Research*.

[21] Bradford M. M. (1976). A rapid and sensitive method for the quantitation of microgram quantities of protein utilizing the principle of protein-dye binding. *Analytical Biochemistry*.

[22] Marklund S., Marklund G. (1974). Involvement of the superoxide anion radical in the autoxidation of pyrogallol and a convenient assay for superoxide dismutase. *European Journal of Biochemistry*.

[23] Aebi H. (1984). Catalase in vitro. *Methods in Enzymology*.

[24] Daigle C. C., Chalupa D. C., Gibb F. R. (2003). Ultrafine particle deposition in humans during rest and exercise. *Inhalation Toxicology*.

[25] Harbison M. L., Brain J. D. (1983). Effects of exercise on particle deposition in Syrian golden hamsters. *American Review of Respiratory Disease*.

[26] Romero S. A., Minson C. T., Halliwill J. R. (2017). The cardiovascular system after exercise. *Journal of Applied Physiology*.

[27] Rodrigues N. A., Torsoni A. S., Fante T., Dos Reis I. G., Gobatto C. A., Manchado-Gobatto F. B. (2017). Lactate minimum underestimates the maximal lactate steady-state in swimming mice. *Applied Physiology, Nutrition, and Metabolism*.

[28] Yoshimura A., Shimomura Y., Murakami T. (1996). Glycogen depletion of the intrafusal fibers in a mouse muscle spindle during prolonged swimming. *American Journal of Physiology*.

[29] Petrosino J. M., Heiss V. J., Maurya S. K. (2016). Graded maximal exercise testing to assess mouse cardio-metabolic phenotypes. *PLoS One*.

[30] Walsh R. C., Koukoulas I., Garnham A., Moseley P. L., Hargreaves M., Febbraio M. A. (2001). Exercise increases serum Hsp72 in humans. *Cell Stress & Chaperones*.

[31] Gordon C. J., Phillips P. M., Beasley T. E. (2016). Pulmonary sensitivity to ozone exposure in sedentary versus chronically trained, female rats. *Inhalation Toxicology*.

[32] Wong C. M., CQ O., Thach T. Q. (2007). Does regular exercise protect against air pollution-associated mortality?. *Preventive Medicine*.

[33] Brioche T., Lemoine-Morel S. (2016). Oxidative stress, sarcopenia, antioxidant strategies and exercise: molecular aspects. *Current Pharmaceutical Design*.

[34] Fiuza-Luces C., Garatachea N., Berger N. A., Lucia A. (2013). Exercise is the real polypill. *Physiology*.

[35] Radons J. (2016). The human HSP70 family of chaperones: where do we stand?. *Cell Stress & Chaperones*.

[36] Fu Q., Levine B. D. (2013). Exercise and the autonomic nervous system. *Handbook of Clinical Neurology*.

[37] Li Z., Song Y., Xing R. (2013). Heat shock protein 70 acts as a potential biomarker for early diagnosis of heart failure. *PLoS One*.

[38] Njemini R., Forti L. N., Mets T. (2017). Sex difference in the heat shock response to high external load resistance training in older humans. *Experimental Gerontology*.

[39] Ye J., Zhu R., He X. (2014). Association of plasma IL-6 and Hsp70 with HRV at different levels of PAHs metabolites. *PLoS One*.

[40] Ludwig M. S., Minguetti-Camara V. C., Heck T. G. (2014). Short-term but not long-term hypoglycaemia enhances plasma levels and hepatic expression of HSP72 in insulin-treated rats: an effect associated with increased IL-6 levels but not with IL-10 or TNF-*α*. *Molecular and Cellular Biochemistry*.

[41] Brook R. D., Rajagopalan S., Pope C. A. (2010). Particulate matter air pollution and cardiovascular disease: an update to the scientific statement from the American Heart Association. *Circulation*.

[42] Yatera K., Hsieh J., Hogg J. C. (2008). Particulate matter air pollution exposure promotes recruitment of monocytes into atherosclerotic plaques. *American Journal of Physiology Heart and Circulatory Physiology*.

[43] Meagher E. A., FitzGerald G. A. (2000). Indices of lipid peroxidation in vivo: strengths and limitations. *Free Radical Biology & Medicine*.

[44] Newby D. E., Mannucci P. M., Tell G. S. (2015). Expert position paper on air pollution and cardiovascular disease. *European Heart Journal*.

[45] Mukhopadhyay I., Nazir A., Saxena D. K., Chowdhuri D. K. (2003). Heat shock response: hsp70 in environmental monitoring. *Journal of Biochemical and Molecular Toxicology*.

[46] Jenei Z. M., Gombos T., Forhecz Z. (2013). Elevated extracellular HSP70 (HSPA1A) level as an independent prognostic marker of mortality in patients with heart failure. *Cell Stress & Chaperones*.

